# Leveraging the bacteria for enhanced cancer immunotherapy: from a perspective of synthetic biology

**DOI:** 10.1080/15384047.2026.2683171

**Published:** 2026-06-04

**Authors:** Xing Liu, Hejin Zhang, Renchun Du, Zixu Liu, Yunyun Ren, Xiao Yang

**Affiliations:** a Center of Stomatology, The Second Affiliated Hospital, Jiangxi Medical College, Nanchang University, Nanchang, People's Republic of China; b Huankui Academy, Nanchang University, Nanchang, Jiangxi, People's Republic of China; c The Second Clinical Medicine School, Nanchang University, Nanchang, People's Republic of China; d The First Clinical Medicine School, Nanchang University, Nanchang, People's Republic of China; e Queen Mary College of Nanchang University, Nanchang, People's Republic of China

**Keywords:** Synthetic biology, bacteria-based immunotherapy, cancer treatment, tumor microenvironment, immune system activation, personalized medicine

## Abstract

In recent years, synthetic biology has been widely applied to engineer and program cellular behaviors. Using this approach, bacteria can be designed to express immunotherapeutic agents, improve tumor targeting, and deliver therapeutic payloads directly to tumor sites. To further improve efficacy, strategies such as hypoxia-responsive promoters, bacterial swarming, and extracellular vesicles (EVs) have been investigated, along with the synergistic effects of combining bacterial therapy with other treatments (e.g., photodynamic therapy, chemotherapy, immune checkpoint inhibitors). This review summarizes recent advances in synthetic biology for bacteria-based cancer immunotherapies, focusing on how bacterial agents activate the immune system and the engineering strategies used to achieve tumor targeting.

## 1. Introduction

Cancer remains a major global health challenge, with malignancies such as lung, gastric, and hepatocellular carcinoma imposing significant clinical burdens.^1^ Although conventional treatments, including surgery, radiotherapy, and cytotoxic chemotherapy, have demonstrated efficacy, their severe side effects often compromise patients’ quality of life.^2^ Therefore, the development of safer and more targeted therapeutic strategies is urgently needed. Among emerging approaches, cancer immunotherapy—particularly chimeric antigen receptor T-cell (CAR-T) therapy—has shown transformative potential by achieving remarkable efficacy in refractory malignancies.^3^ However, tumor immune evasion mechanisms—particularly programmed death-ligand 1 (PD-L1) expression—continue to limit treatment efficacy, underscoring the need for innovative therapeutic strategies.^4^ Recent advances suggest that cytokine modulation and immune checkpoint blockade represent promising approaches to overcome these challenges.^5^

**Figure 1.**
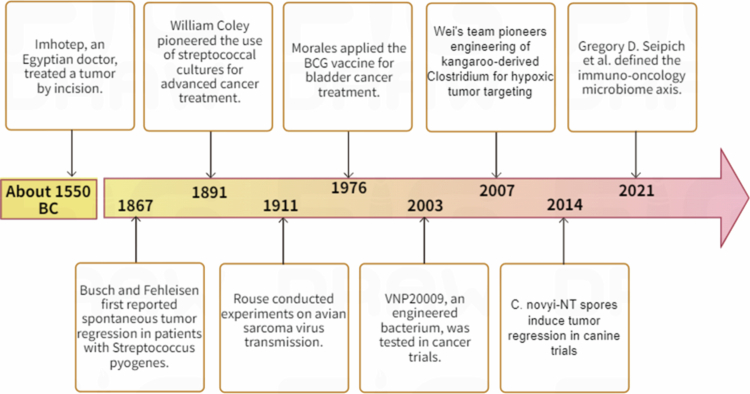
Chronology of major advances in bacteria-based cancer immunotherapy (from ancient times to the present). The timeline highlights pivotal developments, including early clinical observations, the application of bacterial vaccines, the engineering of bacterial vectors, and emerging insights into the immuno-oncology microbiome axis.

Notably, a deeper understanding of the tumor microenvironment (TME) has reinforced bacterial immunotherapy as a promising therapeutic strategy. Compared with conventional treatments, this approach offers superior tumor specificity and reduced systemic toxicity. Its conceptual foundation originates from early observations and has since been refined through advances in microbiology and genetic engineering. Over time, bacterial anticancer strategies have progressed from direct bacterial administration (e.g., Coley’s toxins and the therapeutic use of Bacillus Calmette-Guérin (BCG)) to the precise modulation of intratumoral microbiota using synthetic biology (exemplified by engineered strains such as *Salmonella* VNP20009 and *Clostridium novyi*-NT).^6-11^ Indeed, synthetic biology revolutionizes anticancer therapeutics through the engineering of bacterial vectors to augment specific functions, including targeted payload delivery and immune activation.^12-15^ Engineered bacterial systems have been shown to produce antineoplastic agents in situ, act as biosensors, and serve as targeted delivery platforms.^16^ To illustrate this evolutionary path, Figure 1 provides a chronological overview of pivotal milestones, from empirical beginnings to contemporary engineered approaches, in the development of bacterial immunotherapy.

Specifically, this review elucidates: (i) synthetic biological strategies to enhance bacterial targeting and immunomodulation within the TME; (ii) mechanistic insights into innate and adaptive immune activation including antigen presentation pathways; (iii) therapeutic applications of bacteria-nanomaterial conjugates for precision therapy; and (iv) synergistic potential with photodynamic therapy (PDT), photothermal therapy (PTT), and radiotherapy. Collectively, the integration of synthetic biology with bacterial immunotherapy represents a paradigm-shifting strategy in oncological treatment, potentially transforming the therapeutic landscape against this pervasive disease.

## 2. Effects of bacterial therapeutic agents on immune system

This section focuses on the fundamental immunological mechanisms by which bacterial agents activate host antitumor immunity, providing the mechanistic rationale for the therapeutic strategies detailed in Chapter 4. We first dissect the innate immune pathways triggered by bacterial pathogen-associated molecular patterns and then analyze the subsequent engagement of adaptive immunity, laying the groundwork for understanding their translational applications.

### 2.1. Bacterial PAMPs as activators of antitumor innate immunity

Bacterial immunotherapy exerts its antitumor effects primarily by activating the innate immune system through pathogen-associated molecular patterns (PAMPs). These microbial structures, known as PAMPs, are recognized by specific pattern-recognition receptors (PRRs) on or in immune cells. This recognition initiates downstream signaling pathways that trigger innate immune activation and shape adaptive immunity. Key bacterial PAMPs and their receptors include: lipopolysaccharide (LPS), which is recognized by Toll-like receptor 4 (TLR4)^17^; flagellin, detected by TLR5^18^; unmethylated CpG-rich bacterial DNA, a ligand for TLR9^19^; and peptidoglycan fragments, which activate intracellular nucleotide-binding oligomerization domain-containing protein 1 and 2 (NOD1/2) receptors.^20^ Initial PAMP recognition by PRRs, including TLRs, triggers an immunostimulatory cascade that activates immune cells and induces inflammatory mediator production.^21^ Subsequent studies have shown that PRR engagement recruits adapter proteins such as myeloid differentiation primary response 88 (MyD88) or TIR-domain-containing adapter-inducing interferon-*β* (TRIF) via TIR domain interactions, forming signaling complexes that initiate downstream pathways. MyD88-dependent signaling activates the nuclear factor kappa-light-chain-enhancer of activated B cells (NF-κB) and mitogen-activated protein kinase (MAPK) cascades, driving the expression of pro-inflammatory cytokines, including tumor necrosis factor-*α* (TNF-*α*), interleukin-6 (IL-6), and IL-12. These cytokines activate innate effector cells such as natural killer (NK) cells and macrophages, regulate immune cell recruitment, and amplify inflammatory responses. In contrast, TRIF-dependent signaling induces type I interferon (IFN) production, which directly enhances innate antitumor immunity by augmenting NK cell cytotoxicity and promoting dendritic cell maturation.^22^ Bacterial immunotherapeutic agents serve as potent PAMP delivery systems; a notable example is *Mycobacterium bovis* BCG, a strong TLR2/4/9 agonist widely used in bladder cancer therapy.^23^ A key outcome of PRR activation is the maturation and migration of dendritic cells (DCs), which significantly improves their ability to secrete immunostimulatory cytokines and activate innate lymphocytes such as NK cells, thereby potentiating early innate antitumor responses.^24^ ^,^ ^25^ Collectively, these mechanisms not only amplify innate immune responses but also provide essential signals for initiating adaptive immunity.

### 2.2. Harnessing adaptive immunity for antitumor responses

This section aims to delineate the mechanisms by which bacterial immunotherapy mobilizes the adaptive immune system to achieve targeted and durable anticancer effects. The discussion is structured around three key processes: antigen-specific T cell activation, immune memory formation, and the strategic use of combination therapies to amplify these effects. These mechanistic principles are later manifested in specific therapeutic designs, such as engineering bacteria to deliver tumor-associated antigens or immunomodulatory fusion proteins, as will be explored in Chapter 4.

Beyond activating innate immunity, bacterial immunotherapeutic agents also engage adaptive responses to achieve tumor-specific effects. A key event in this process is the activation of DCs, which bridge innate and adaptive immunity. Upon encountering bacteria, DCs present antigens on major histocompatibility complex (MHC) molecules to T cells. These antigens include tumor-associated or tumor-specific antigens expressed by malignant cells, as well as bacterial antigens. Potent bacterial PAMPs such as flagellin (FlaB) serve as potent immune stimulants. For instance, flagellin signaling through TLR5 promotes dendritic cell maturation and shapes the adaptive immune response, with the Th polarization direction being context-dependent and influenced by the local cytokine milieu. This activation of antigen-presenting cells (APCs) leads to the upregulation of co-stimulatory molecules and enhanced antigen presentation. Consequently, this elicits robust T-cell responses directed against the bacterial vector. Through mechanisms such as epitope spreading or bystander effects, this activated immune state can be redirected against the tumor, linking bacterial immune stimulation to antitumor immunity.^24^ To exploit this, engineered bacteria are often designed to deliver defined tumor antigens. A notable example is attenuated *Listeria monocytogenes* expressing the human papillomavirus (HPV) E7 protein for cervical cancer therapy.^26^ This strategy activates cytotoxic T lymphocytes (CTLs), enabling tumor cell recognition and elimination.^27^ Consequently, the degree of CTL infiltration within tumors is considered a strong prognostic indicator.

Bacterial agents also modulate helper T-cell subsets, notably Th1 and T helper 17 (Th17) cells, inducing the secretion of cytokines such as IFN-*γ* and IL-17, which enhance inflammatory responses and immune surveillance.^28^ Among these, IFN-*γ* plays a pivotal role by promoting antigen presentation and upregulating MHC expression on tumor cells, thereby exposing additional tumor-associated antigens (TAAs) and tumor-specific antigens (TSAs) for CTL recognition.^29^ Furthermore, bacterial immunotherapy drives the formation of immune memory, providing rapid and durable protection against tumor recurrence. To amplify these adaptive immune responses, combination strategies are actively explored. These include synthetic TLR agonizts (e.g., monophosphoryl lipid A [TLR4], CpG oligodeoxynucleotides [TLR9]) and stimulator of interferon genes (STING) agonizts (e.g., cGAMP derivatives, DMXAA analogs), which synergize with bacterial PAMPs to amplify DC activation, cytokine production, and T-cell priming.^30^ ^,^ ^31^ For instance, certain intracellular bacteria such as Listeria monocytogenes naturally produce cyclic di-AMP (c-di-AMP), a potent STING agonist that cooperates with bacterial PAMPs to enhance type I interferon production and antitumor T-cell responses.^32^ Beyond these direct PAMP-receptor interactions, bacterial components can also reshape the immunological set point of the TME to potentiate adaptive immunity. For example, gut-derived LPS remodels the pancreatic tumor microenvironment by stimulating T cell infiltration via the TLR4/MyD88/AKT/NF-κB signaling cascade. This LPS-driven pathway transcriptionally upregulates PD-L1 expression on tumor cells, thereby creating a condition in which PD-L1 checkpoint blockade synergizes with the remodeled immune context to suppress tumor growth.^33^

**Figure 2.**
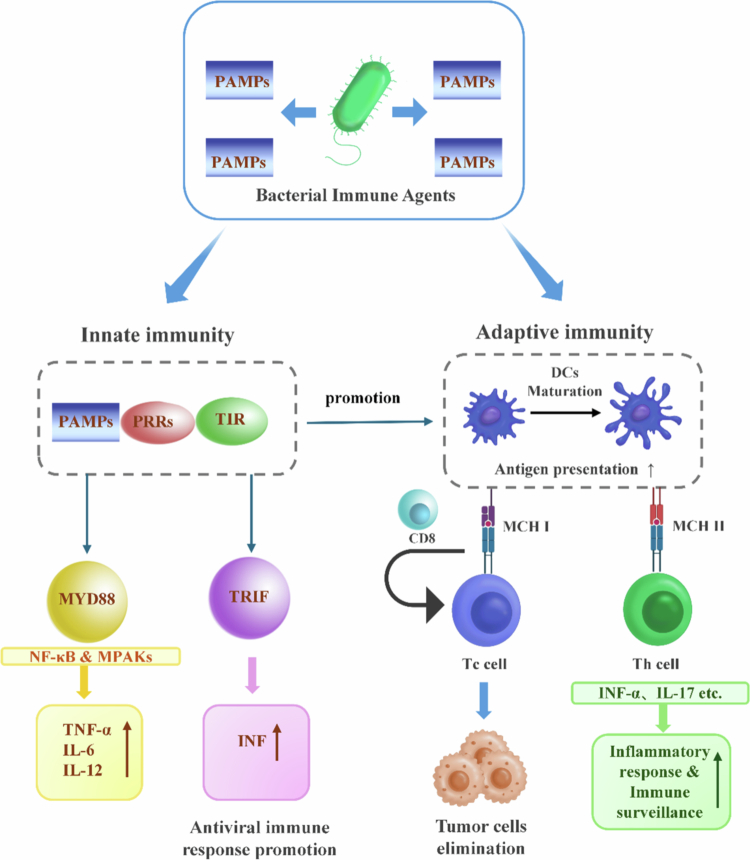
Mechanism of innate and adaptive immune activation by bacterial therapeutic agents. Left Panel (Innate Immunity Activation by Bacterial Agents): Bacterial-derived PAMPs engage host PRRs, initiating tumor-focused immunostimulatory cascades. The recruitment of adapter proteins (MyD88, TRIF) activates transcription factors (NF-κB, MAPKs), leading to the production of pivotal mediators: pro-inflammatory cytokines (TNF-*α*, IL-6, IL-12) and type I interferons (IFN-α/β). These signals collectively establish an inflammatory antitumor microenvironment and critically drive the maturation of DCs, a key prerequisite for adaptive immunity. Right Panel (Adaptive Immunity Engagement and Effector Phase): Mature DCs, activated by bacterial components, bridge innate and adaptive immunity by presenting processed tumor and bacterial antigens. This primes cytotoxic CD8⁺ T cells (CTLs) for direct tumor cell killing and activates helper T cell subsets (Th1, Th17). A key effector cytokine, IFN-*γ*, produced by bacteria-activated Th1 cells, feedbacks to enhance tumor antigen visibility by upregulating MHC expression on tumor cells. The synergistic action of CTLs and helper T cells, initiated and amplified by bacterial immunotherapy, results in targeted tumor cell elimination and the establishment of long-term antitumor immune memory.

In summary, bacterial immunotherapy activates multiple adaptive immune components through a multi-tiered mechanism (Figure 2). This strategy leverages both the intrinsic immunogenicity of bacteria and the synergistic potential of immunotherapeutic agents and small-molecule modulators, forming a robust conceptual foundation for next-generation cancer immunotherapy.

## 3. The production of bacteria-based therapeutic agents

**Figure 3.**
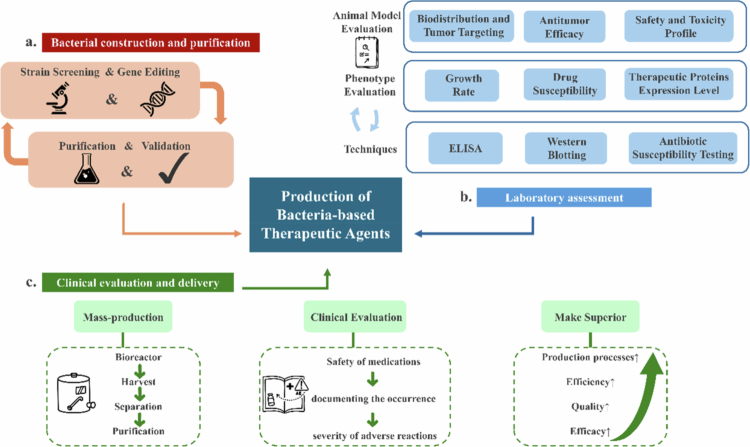
Schematic diagram of the process flow of bacterial immune preparations from bacterial construction to clinical application. The workflow outlines three consecutive stages: (a) Bacterial construction and purification, involving strain selection, genetic engineering, and clonal validation; (b) Comprehensive laboratory assessment, encompassing in vitro Phenotype Evaluation and essential in vivo Animal Model Evaluation of biodistribution, antitumor efficacy, and safety; and (c) Clinical evaluation and delivery, which includes scaled cGMP production, phased clinical trials, and iterative process optimization.

The development of contemporary bacterial immunotherapeutics from discovery to clinical application follows a defined translational pipeline, as illustrated in Figure 3. This multistage workflow encompasses the rational selection and engineering of safe, tumor-targeting bacterial chassis, rigorous preclinical and clinical validation, and ultimately, scalable current Good Manufacturing Practice (cGMP) production.

The ideal bacterial chassis must exhibit low pathogenicity, a favorable safety profile, and intrinsic tumor tropism. Common candidates include attenuated strains of *Salmonella spp.*, *Escherichia coli*, and *Listeria monocytogenes.* ^24^ ^,^ ^34^ ^,^ ^35^ Their genetic engineering relies on versatile vector systems. Beyond synthetic plasmids, the strategic engineering of a bacterium’s native (cryptic) plasmids offers advantages in host compatibility and stable, antibiotic-free maintenance. For stable genomic integration, plasmid-based homologous recombination remains a fundamental strategy.

Precise genetic modifications employ both traditional and advanced genome-editing technologies. While the clustered regularly interspaced short palindromic repeats (CRISPR)/Cas9 is widely used for targeted gene knockout, the CRISPR/Cas12a platform has emerged as a potent alternative for multiplexed editing. Cas12a’s ability to process its own guide RNA arrays facilitates simultaneous regulation of multiple targets. For instance, the dCas12a-based “MultiduBE” system enables multiplexed base editing to drive complex metabolic reprogramming in bacteria.^36^ Concurrently, optimizing transgene expression is critical. Codon optimization has evolved into sophisticated strategies that balance codon preference with translation kinetics and protein folding. Advanced deep learning models now intelligently design coding sequences to maximize heterologous protein expression in bacterial chassis like *E. coli*, significantly improving therapeutic protein yield.^37^

Engineered strains undergo purification to confirm genotypic stability and phenotypic consistency.^38^ Concurrently, genotypic verification is performed using PCR or Sanger sequencing.^39^ Phenotypic attributes—including antibiotic susceptibility, growth kinetics, and therapeutic protein expression—are characterized via disc diffusion assays, ELIZA, or immunoblotting. To improve bacterial stability and functionality, nanoparticle encapsulation strategies have been explored. For instance, liposomal coatings enhance resistance to gastric acids, while gold nanoparticle conjugation improves photoacoustic imaging and bactericidal properties.^40^

Upon successful preclinical validation, therapeutic batches are produced to meet clinical demand. This bioprocessing pipeline includes bioreactor cultivation, biomass harvesting, purification, and final formulation. Techniques such as hollow-fiber bioreactors (HFBs), continuous centrifugation, tangential flow filtration, and affinity chromatography are implemented under Good Manufacturing Practice (GMP) standards.^41^ Subsequently, phased clinical trials—from preclinical studies through phase III—are conducted to assess pharmacokinetics, safety, and efficacy, representing a critical step toward clinical translation.^42^ After regulatory approval, manufacturing processes are iteratively refined to increase yield, reduce production costs, enhance product quality, and explore novel therapeutic applications.

Advances in synthetic biology and nanotechnology have transformed the landscape of scalable bacterial production, enabling innovative strategies for next-generation immunotherapies.

## 4. Strategies of bacteria-based cancer immunotherapy

Building upon the fundamental immunological mechanisms described in Chapter 2, this chapter explores their translational application. We categorize the diverse bacteria-based anticancer strategies into distinct therapeutic paradigms and detail their design, delivery, and clinical potential.

**Figure 4.**
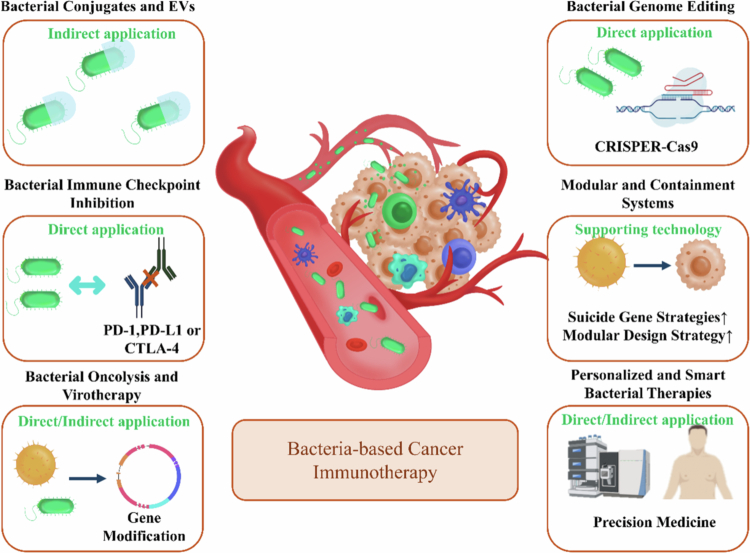
Schematic representation of strategies in bacteria-based cancer immunotherapy. Direct application refers to the use of live bacteria (wild‑type or engineered) as intratumoral agents that locally deliver therapeutics, induce oncolysis, or modulate the tumor microenvironment. Indirect application refers to the use of bacterial components (e.g., outer membrane vesicles, attenuated strains) or bioengineered bacteria as ex vivo production platforms for vaccines, viral vectors, or immunostimulatory molecules, which are then administered separately. Supporting technology denotes modular design and biocontainment systems that enable safe and controllable therapeutic delivery.

Bacteria-based anticancer strategies can be broadly categorized into two distinct paradigms based on their mode of application: direct and indirect use. Direct strategies employ live bacteria—either wild-type (WT) or engineered—as intratumoral agents that locally deliver therapeutics, induce oncolysis, or modulate the TME. Indirect strategies utilize bacterial components (e.g., outer membrane vesicles, attenuated strains) or bioengineered bacteria as platforms for producing vaccines, viral vectors, or immunostimulatory molecules ex vivo, which are then administered separately. The following sections delineate these strategies, with their key features summarized in Figure 4.

### 4.1. Engineered tumor tropism via synthetic biology

As a direct application strategy, bacterial tumor tropism can be classified into two categories: non-engineered (native) tropism and engineered tropism. Non-engineered tropism relies on the inherent biological properties of certain bacterial species—such as the facultative anaerobic metabolism of *Escherichia coli* and *Salmonella* spp. that drives colonization of hypoxic tumor cores, or native chemotaxis toward tumor-derived metabolites. In contrast, synthetically engineered bacteria exhibit enhanced or entirely novel tumor tropism achieved through genetic modification, enabling targeted delivery of immunotherapeutic agents in response to programmed chemotactic cues within the TME. This selective targeting reduces off-tumor cytotoxicity. Below, we first discuss native tropism mechanisms shared by many bacterial chassis, then detail how synthetic biology further augments these capabilities.

Bacterial accumulation in tumors occurs via both passive and active mechanisms. Passive targeting is mediated by the enhanced permeability and retention (EPR) effect in leaky tumor vasculature, whereas active tropism is driven by engineered or native responses to hypoxia, necrosis, and metabolic gradients, as well as engineered sensing circuits.^43^ Importantly, the role of native tumor-associated bacteria (TAB) within tumors is complex and context-dependent. Certain species, such as *Fusobacterium nucleatum* in colorectal cancer and *Pseudomonas aeruginosa* in lung cancer, have been shown to actively promote tumor progression. They achieve this through mechanisms like forming protective biofilms, secreting metabolites (e.g., iron-scavenging pyoverdine) that shield tumor cells from ferroptosis, and inducing epithelial-mesenchymal transition.^44^ This dual nature—being both a component of the TME and a potential driver of malignancy—highlights the significance of understanding native bacteriotropism.

While the above mechanisms represent inherent bacterial properties, synthetic biology enables the rational design and augmentation of tumor tropism. Synthetic biology enables the design of bacteria that respond to TME-specific signals, including acidic pH and tumor-derived metabolites. Hypoxia, a hallmark of the TME, drives metabolic reprogramming, invasion, metastasis, radioresistance, and immune evasion by reducing tumor immunogenicity.^45^ While facultative anaerobes such as *Escherichia coli* Nissle 1917 (EcN) and *Salmonella* spp. naturally colonize hypoxic tumor regions via their inherent metabolic versatility, synthetic engineering efforts have primarily focused on rewiring bacteria to sense and respond to other TME cues that are not naturally exploited. Furthermore, engineering often co-opts native TAB strategies. For instance, bacteria can be designed to sense and respond to the acidic, lactate-rich TME, restricting therapeutic gene expression to tumors for enhanced specificity.^46^ Bacterial translocation into tumor tissue is further facilitated by motility systems, including flagella and type IV pili, as well as chemoreceptor-mediated taxis.

Genetic engineering can further enhance tumor tropism by endowing bacteria with synthetic adhesion properties. Piñero-Lambea et al. developed a modular synthetic adhesin (SA) platform in which the immunoglobulin-like domains of the native Intimin adhesin from enteropathogenic *Escherichia coli* were replaced with single-domain nanobodies (VHHs). By selecting nanobodies that recognize tumor-associated antigens such as HER2, the engineered bacteria acquired de novo tumor-specific adhesion and colonized solid tumors expressing the cognate antigen with significantly greater efficiency than their non-adherent counterparts.^47^ Complementing adhesion-based targeting, bacteria can also be equipped with engineered sensing systems to detect metabolic gradients within solid tumors. Panteli and Forbes demonstrated that *E. coli* engineered to express a glucose-sensing chemotaxis receptor (Trz1) can detect spatial profiles in glucose concentration within solid tumor cell masses, enabling the bacteria to actively track sugar gradients generated by metabolically heterogeneous tumor regions—a capability that passive diffusion alone cannot achieve.^48^ Additionally, tumor cells undergo aerobic glycolysis, producing lactate that acidifies the TME. This acidic environment, potentiated by histone lysine lactylation (Kla), promotes tumor progression.^49^ Acid-responsive bacterial systems have therefore been engineered to secrete enzyme inhibitors or lysins, modulating TME biochemistry and impairing tumor viability.^50^

Beyond environmental responsiveness, bacterial swarming behavior (BSB), exemplified by *Pseudomonas aeruginosa*, provides a motility mechanism for deep tumor penetration. During swarming, *P. aeruginosa* forms multicellular rafts that migrate rapidly across surfaces via flagellar propulsion, generating complex vortical patterns.^51^ In cancer applications, this native swarming capability has been harnessed by engineered *Salmonella* flagellar systems to enhance intratumoral infiltration and replication, creating novel therapeutic opportunities.^52^ Clinical translation, however, requires a detailed understanding of swarming dynamics, including fission–fusion kinetics during collective migration. To this end, chemotactic behaviors are being reprogrammed to optimize tumor-directed migration, while small-molecule inhibitors of flagellin biosynthesis or c-di-GMP signaling are employed to spatially constrain bacterial dissemination.^53^

Overall, the interplay between native bacterial tropism and synthetic reengineering—from exploiting inherent anaerobic metabolism to programming novel sensing circuits and swarming mechanisms—enables precise therapeutic delivery while minimizing collateral tissue damage.

### 4.2. Targeted cancer therapies: bacterial conjugates and EVs

An alternative strategy for tumor-specific delivery employs bacteria–nanoparticle conjugates and bacterial extracellular vesicles (BEVs) as engineered platforms. These systems synergize with tropic bacteria to enhance targeting precision and primarily represent an indirect application paradigm, wherein bacterial components or engineered vectors are produced ex vivo and administered, rather than relying on intratumoral bacterial colonization.

Bacteria–nanoparticle conjugates integrate principles of synthetic biology and nanomedicine, leveraging bacterial tropism for site-specific nanocarrier deposition. Nanoparticle (NP) properties—including size, morphology, surface charge, and material composition—can be tuned to optimize drug loading, release kinetics, and biocompatibility. For example, colorectal cancer membrane-coated mesoporous silica NPs (Mel-SiO₂@CCM) exploit homotypic adhesion for tumor targeting and Gal-GalNAc-Fap2 interactions for selective *Fusobacterium nucleatum* elimination, achieving 91% tumor suppression via dual bacteriolytic and immunomodulatory effects.^54^ Functionalized NPs enable spatiotemporal delivery of diverse payloads, including chemotherapeutics and siRNAs. Flagella-drug nanoconjugates (FDNCs), for instance, utilize flagellar immunogenicity to enhance cellular uptake and acid-labile linkers for triggered release.^55^ NP selection depends on therapeutic goals: magnetic NPs facilitate magnetothermal therapy, gold NPs enable photothermal therapy through efficient photoconversion, and silica NPs serve as robust delivery vehicles.^56-58^ Mechanistically, these conjugates mediate direct tumoricidal effects, immune activation, angiogenesis inhibition, and enhanced intratumoral drug accumulation. Despite these advantages, translational challenges remain. Key obstacles include biosafety concerns, immune clearance, optimization of NP pharmacokinetics, and scalable manufacturing.

Concurrently, bacterial extracellular vesicles (BEVs) have emerged as promising nanocarriers due to their inherent biocompatibility, stability, and tumor-homing capabilities. These lipid-bilayer vesicles mediate intercellular communication by transporting proteins, nucleic acids, and metabolites.^59^ BEVs can be internalized by tumor cells through endocytosis or membrane fusion, enabling efficient payload delivery.^60^ For example, *Bifidobacterium bifidum* BEVs intrinsically inhibit tumor growth while delivering therapeutic agents against triple-negative breast cancer.^61^ Similarly, *Lactobacillus*-derived BEVs act as immunotherapeutic vectors by transporting immunostimulants and repolarizing macrophages toward antitumor phenotypes.^62^ To further enhance the performance of conjugates and BEVs, surface functionalization strategies—including covalent conjugation or physical adsorption—attach targeting ligands such as peptides or antibodies to nanoparticle or BEV membranes, thereby improving tumor specificity and therapeutic payload delivery.^63^

In summary, although bacteria–NP conjugates and BEVs exhibit considerable therapeutic potential, their clinical translation requires addressing biosafety concerns, manufacturing complexity, and pharmacokinetic optimization to achieve reproducible, tumor-specific efficacy.

### 4.3. Specific applications of synthetic biology: from gene editing to immune regulation

This direct application strategy employs live, engineered bacterial vectors capable of colonizing tumors and performing localized genome editing or immune modulation within the TME. Bacterial genome editing is a key tool for programming bacteria to deliver tumor-specific antigens or immunomodulators. Among available technologies, the CRISPR–Cas system stands out for its high efficiency and multiplexed targeting; however, its application is constrained by the requirement for protospacer-adjacent motifs (PAMs) and the persistent risk of off-target effects. To improve CRISPR–Cas9 specificity, strategies such as guide RNA (gRNA) design optimization, Cas9 protein engineering, and delivery system innovations are actively pursued.^64^ Complementing these genomic modifications, synthetic biology circuits increasingly incorporate tunable gene-expression modules. Gene silencing tools—such as CRISPR interference (CRISPRi), transcription activator-like effector nucleases (TALENs), and RNA interference (RNAi)—allow precise knockdown of target genes, though they may produce transient effects and off-target transcript degradation. Conversely, gene overexpression systems enhance production of immunostimulatory cytokines but face challenges in achieving precise temporal control within the TME. For instance, Mansurov et al. demonstrated that engineered bacteria can be programmed to overexpress cytokines such as interleukin-12 (IL-12) locally, maximizing antitumor effects while minimizing systemic toxicity.^65^

Beyond genome manipulation, live bacterial vectors can modulate immune responses within the TME in a context-dependent manner. These constructs induce secretion of cytokines, including IL-2, TNF-*α*, and IFN-*γ*, activating immune effectors and potentiating tumor eradication. For example, IL-12-expressing *Salmonella* typhimurium stimulates Th1-polarized immunity and enhances CTL activity.^21^ Similarly, attenuated *Salmonella* downregulates epithelial cell adhesion molecule (EpCAM) via AKT/mTOR signaling to suppress metastasis, while poly-arginine-coated antigen delivery establishes antigen-specific CD8+ T cell immunity, synergistically augmented by type I interferon adjuvants.^66^ Engineered *E. coli* Nissle 1917 also colonizes tumors, converts ammonia to L-arginine, delivers PD-L1-blocking antibodies, and enhances immune-mediated clearance.^67^

Critically, synthetic gene circuits enable spatiotemporal control of bacterial viability and therapeutic output, ensuring localized cytokine production and subsequent clearance. These feedback-regulated systems respond to multiple TME cues. Despite these advances, suboptimal antigen presentation efficiency remains a challenge, motivating the development of dynamic regulatory circuits to enhance CD4⁺/CD8⁺ T cell cooperation in tumor elimination. Notably, sialic acid-binding immunoglobulin-type lectin G (Siglec-G) modulates dendritic cell-mediated CD8⁺ T cell priming, and strategic manipulation of this pathway could amplify antigen presentation and improve vaccine efficacy.^68^ Concurrently, bacterially delivered immune checkpoint inhibitors (ICIs) targeting programmed cell death protein 1 (PD-1), PD-L1, and CTLA-4 have demonstrated therapeutic potential.^69^ To reduce immune-related adverse events (irAEs) associated with systemic monoclonal antibodies, engineered bacteria can serve as localized ICI delivery platforms. For example, Shapiro et al. developed a thermally responsive bacterial strain that produces anti-PD-L1 and anti-CTLA-4 antibodies exclusively within tumors.^70^

Extending beyond local delivery, engineered bacteria have been developed as systemic anticancer vaccines. By leveraging their intrinsic PAMPs as built-in adjuvants, bacterial vectors deliver tumor antigens to APCs and elicit durable T cell immunity—addressing a key limitation of conventional peptide vaccines that often lack sufficient immunogenicity.^71^ Clinically, the most advanced example is ADXS11-001, a live-attenuated Listeria monocytogenes secreting an HPV E7-LLO fusion protein, which achieved objective tumor responses in a randomized Phase II cervical cancer trial.^72^ More recently, Redenti et al. engineered the probiotic *Escherichia coli* Nissle 1917 to deliver arrays of patient-specific neoantigens, eliciting potent CD4⁺ and CD8⁺ T cell responses that controlled established primary and metastatic tumors and synergized with anti-PD-L1 blockade in preclinical models.^73^

Collectively, synthetic biology advances have revolutionized both genome editing and immunomodulatory strategies, enabling safer, more effective anticancer therapies.

### 4.4. Bacterial oncolysis and virotherapy

Tumor-selective oncolytic bacteria and viruses represent emerging therapeutic modalities that exploit pathogen tropism to selectively destroy malignant cells. This section is organized into two complementary strategies: direct bacterial oncolysis, and the integration of bacteria with oncolytic virotherapy, including bacteria-mediated viral delivery and direct viral oncolysis.

#### 4.4.1. Direct bacterial oncolysis

Oncolytic bacteria employ multiple mechanisms for tumor-specific translocation, including vascular extravasation, interstitial migration, and direct cellular invasion. Synthetically engineered Salmonella YB1, an oxygen-sensitive attenuated strain, suppresses metastasis through an IFN-*γ*- and NK cell-dependent immune mechanism, in which NK cell-derived IFN-*γ* promotes the accumulation, activation, and cytotoxicity of NK cells against metastatic cancer cells, while *Clostridium* spp. lyse tumor cells via exotoxin release, recruiting immune effectors and enhancing antitumor immunity.^74^ ^,^ ^75^ Mechanistically, *C. difficile* exotoxins A and B mediate distinct effects: toxin A increases vascular permeability to facilitate immune cell infiltration, whereas toxin B binds cell surface receptors, inducing apoptosis.^76^ Additionally, *Salmonella* and *E. coli* engineered to synthesize and locally release cytotoxic payloads (e.g., small molecules, immunotoxins) profoundly reshape the TME. Notably, Wang et al. paraformaldehyde-fixed *Salmonella* coated with manganese dioxide to activate innate immune pathways, achieving abscopal antitumor effects.^77^ Consistent with the ability of *Clostridium* to thrive in hypoxic tumor cores, the attenuated strain C. novyi-NT has demonstrated promising clinical activity when combined with immune checkpoint blockade; a recent Phase Ib trial of intratumoral C. novyi-NT plus pembrolizumab in patients with treatment-refractory solid tumors reported a confirmed objective response rate of 25% with a median duration of response of 10.93 months and stable disease in 69% of patients.^78^ Collectively, these direct oncolytic strategies leverage the intrinsic tumor-homing and tumor-lytic capabilities of bacteria to achieve localized antitumor effects, and they provide the foundational principles for more sophisticated combination approaches discussed below.

#### 4.4.2. Bacteria-mediated viral delivery and direct virotherapy

Beyond direct bacterial oncolysis, a rapidly evolving strategy harnesses bacterial tumor tropism to overcome the pharmacokinetic limitations that hamper conventional virotherapy, such as rapid systemic clearance and poor tumor penetration. By functioning as delivery vehicles for oncolytic viruses or their genetic material, bacteria combine their intrinsic tumor-targeting capabilities with the potent oncolytic and immunostimulatory properties of viruses.

The covalently attached programmable polysaccharide-based encapsulation of *Salmonella* for intracellular delivery (CAPPSID) platform exemplifies innovative delivery strategies, using engineered *Salmonella* typhimurium to transport oncolytic Senecavirus A RNA directly into tumor cells, where bacterial lysis releases the viral genome, launching a potent oncolytic viral infection that can bypass circulating antiviral antibodies.^79^ Similarly, Recent advances include “Virus-Delivering *Salmonella*” (VDS) strains designed to protect and deliver the genome of oncolytic viruses such as the minute virus of mice (MVMp) into cancer cells, leading to successful intratumoral viral propagation and potent bystander effects.^80^

Furthermore, oncolytic virotherapy exploits viral tropism to selectively infect and lyse malignant cells. Contemporary research demonstrates that oncolytic viruses not only induce direct oncolysis but also elicit systemic antitumor immunity—an immunostimulatory effect that is further potentiated when combined with immune checkpoint inhibitors or tumor antigen vaccines.^81^ Building upon these intrinsic properties, oncolytic viruses can be directly employed or genetically engineered for tumor-selective destruction, representing a complementary strategy within the virotherapy paradigm. Viral therapy primarily utilizes the natural properties of viruses or genetic engineering for targeted infection and lysis of tumor cells. Modified adenovirus and herpes simplex virus (HSV) strains have shown significant efficacy in tumor therapy by replicating within tumor cells, inducing cell lysis, and stimulating the body's immune memory.^82^ ^,^ ^83^ Moreover, synthetic biology tools have been employed to design genetic circuits capable of sensing and responding to the TME.^84^ This capability enables on-demand control of viral activity and autonomous payload release. Furthermore, viral therapies also enhance tumor killing by delivering therapeutic genes or immune-stimulating molecules that activate or enhance the host's immune system and strengthen the immune response to the tumor.^85^

In summary, the integration of bacterial delivery platforms with oncolytic virotherapy—whether through bacteria-mediated viral genome delivery or complementary direct viral oncolysis—represents a powerful convergence of two therapeutic modalities. This combined approach exploits the tumor-homing precision of engineered bacteria and the potent lytic and immunogenic properties of oncolytic viruses, offering synergistic potential for next-generation cancer immunotherapy.

### 4.5. Modular and containment systems in bacterial therapies

Implementation of modular design and biocontainment systems is critical for developing safe and effective bacterial immunotherapies. These foundational technologies provide essential safety and control mechanisms for both direct and indirect therapeutic strategies, ensuring precise spatial and temporal regulation of therapeutic activity regardless of the delivery mode. Biocontainment restricts bacterial proliferation and dissemination to prevent off-tumor toxicity. For example, leucine-auxotrophic *Salmonella* typhimurium A1-R requires exogenous leucine for viability, limiting its expansion in vivo.^86^ Suicide gene systems offer density-dependent control, triggering autolysis at threshold populations to maintain ecological equilibrium. Din et al. ’s synchronized lysis circuit (SLC) exemplifies this approach: bacterial quorum sensing induces lysis factor production, releasing therapeutics while regulating population dynamics.^87^

The modular design strategy enhances the flexibility and controllability of therapeutics by incorporating various functional modules, including targeting, release, and safety. This design paradigm enables the customization of bacterial behavior to meet specific therapeutic needs, thereby improving the precision and safety of treatment strategies. Targeting modules primarily modify bacterial surface proteins or ligands to enable selective binding to tumor cells. For instance, Massa et al. enhanced bacterial aggregation at tumor sites and improved invasiveness against CD20+ lymphomas by conjugating an anti-CD20 antibody to the surface of *Salmonella.* ^88^ Similarly, Park et al. endowed bacteria with the ability to specifically bind overexpressed αvβ3 integrins by incorporating an arginine-glycine-aspartate (RGD) peptide into the bacterial outer membrane protein A, resulting in significant targeted anti-tumor effects in xenograft models of melanoma and breast cancer.^89^ The release module controls the timing and dosage of the therapeutic agent delivered by the bacteria, utilizing the bacteria's natural secretory pathways, such as the type III secretion system (T3SS), to efficiently target the host cell membrane or cytoplasm and activate specific immune responses. The safety module, on the other hand, consists of suicide circuits or externally controlled kill switches to ensure the safety of the treatment process. Two main types of kill switches have been identified: “dead man” kill switches and “codon” kill switches. The “dead man” lethal switch maintains cell viability through unbalanced transcriptional repression in combination with specific input signals, whereas the “codon” lethal switch employs a two-tiered transcriptional structure that combines a set of hybrid LacI-GalR transcription factors to regulate bacterial survival through a triad of different signals.^90^

Integration of these modules ensures precise, contained therapeutic action.

### 4.6. Personalized and smart bacterial therapies

Microbiome-informed personalized therapies and environmentally responsive “smart” bacteria represent advanced translational paradigms. These approaches strategically leverage both direct intratumoral mechanisms and indirect systemic effects to achieve precision oncology goals.Tumor–microbiome interactions significantly influence carcinogenesis, treatment response, and immunotherapy efficacy. Rare commensal strains may serve as novel biotherapeutics, and patient-specific microbiota profiling can guide the selection of therapeutic bacteria. For example, genetically modified gut commensals have been engineered to release anticancer agents within the TME. Tanoue et al. demonstrated that defined *Listeria* consortia enhance host resistance and potentiate ICI efficacy.^91^ Intratumoral microbiota also modulate chemoresistance; for instance, *Fusobacterium* spp. confer gemcitabine resistance.^92^ Currently, personalized therapies aim to improve immune responses against specific tumors by leveraging the patient’s microbiome, such as using probiotic strains engineered to deliver antitumor drugs.

Smart bacterial therapies utilize engineered bacteria capable of sensing specific TME signals to selectively release therapeutic agents—including chemotherapeutics, immunostimulatory factors, or lysogenic viruses. This on-demand release minimizes systemic toxicity and enhances therapeutic efficacy. Therapeutic precision is further improved when these bacteria are combined with nanocarriers, enabling higher drug concentrations at tumor sites.^93^

Although clinically nascent, these strategies offer transformative potential for precision oncology.

## 5. Development of bacteria-based combination treatment

**Figure 5.**
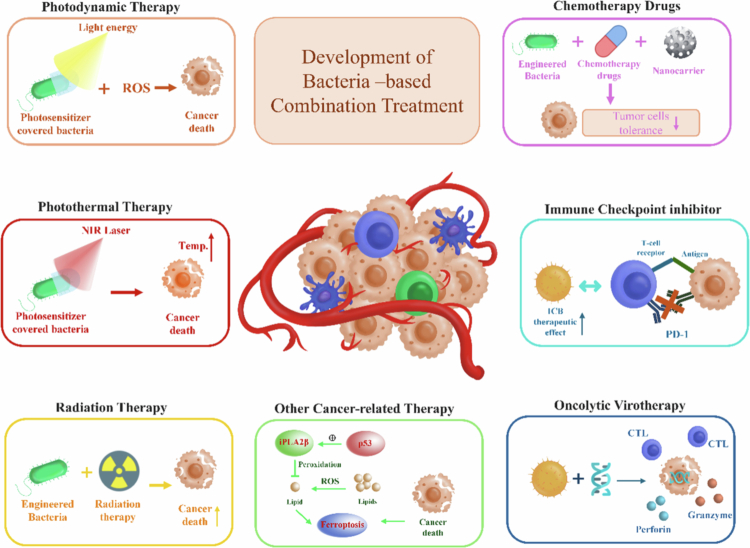
Overview of integrated bacterial therapies in cancer treatment, combining photodynamic, photothermal, chemotherapy, radiation, immune checkpoint, and virotherapy approaches.

Multi-mechanism immunotherapy has emerged as a superior strategy for tumor. Although conventional modalities—including chemotherapy, radiotherapy, and immunotherapy—remain therapeutic cornerstones, clinical evidence demonstrates suboptimal efficacy of monotherapies.^94^ Similarly, standalone bacterial immunotherapy rarely achieves complete tumor clearance. Consequently, bacterially-mediated synergistic regimens are being actively explored. Herein, we elucidate prominent combination strategies, as summarized in Table 1 and illustrated in Figure 5.

**Table 1.** Summary of Bacteria-Based Combination Therapies for Cancer Treatment.

Treatment Type	Definition	Advantages (Single Therapy)	Disadvantages (Single Therapy)	Enhancement with Bacterial Immunotherapy	Ref
Photodynamic therapy(PDT)	Uses photosensitizers to generate ROS in the presence of light	Enhances tumor targeting, activates immune system	Limited by distribution, light penetration, oxygen availability	Engineered bioluminescent *Salmonella* (Luc-S.T.ΔppGpp) overcoming penetration limits for opaque/large tumors with elicited immunity.	[95]
Photothermal therapy(PTT)	Converts light energy to heat to destroy tumor cells	Non-invasive, precise targeting, minimal side effects	PTA aggregation, tissue penetration, oxygen availability	PDA-coated *E. coli* Nissle 1917 delivering metformin reverses PDT-induced PD-L1 upregulation.	[96]
Radiation therapy(RT)	Uses high-energy rays to induce DNA damage in cancer cells	Targets specific tumor regions, overcomes monotherapy limitations	May inadvertently injure surrounding healthy tissues, resulting in toxicity	Bacterial cellulose-immobilized ^131^I-αPD-L1 enables sustained radio-immunotherapy, suppressing metastasis via enhanced immunogenic cell death.	[97]
Chemotherapy drugs	Uses chemical agents to kill rapidly dividing cells	Improves drug targeting, enhances immunotherapeutic effects	Need to optimize stability, targeting, therapeutic efficiency	Facultative anaerobes exploit hypoxic TME for localized drug delivery. Improved targeting, reduction of side effects.	[34]
Immune checkpoint inhibitors	Targets immunomodulatory molecules to restore T-cell activity	Enhances antitumor immune responses	Limited response rates, potential adverse events	Engineered bacteria with lysis circuit locally release PD-L1/CTLA-4 nanobodies, enhancing systemic antitumor immunity via single injection.	[98]
Oncolytic virotherapy(OVT)	Viruses infect and selectively eliminate tumor cells	Directly destroys tumor cells, triggers systemic antitumor immune response	Control of viral dissemination, avoiding harm to normal tissues	*MV-eGFP-Pwt* armed with *P* gene enhances IFN evasion for improved oncolysis.	[99]

### 5.1. Combination with photodynamic therapy

Photodynamic therapy (PDT) employs photosensitizers to generate cytotoxic reactive oxygen species (ROS) upon oxygen-mediated photoactivation.Despite its tissue-sparing advantage, PDT efficacy is constrained by suboptimal photosensitizer biodistribution, limited light penetration, and tumor hypoxia. Bacterially-mediated delivery systems precisely overcome these limitations. Notably, Guo et al. utilized *Salmonella* expressing fluorogen-activating protein (FAP) for tumoral targeting; FAP complexation with fluorogens generated ROS, inducing oxidative stress-mediated apoptosis while stimulating pro-inflammatory cytokine production.^100^ Furthermore, *Porphyromonas gingivalis (Pg)* functioned as a potent endogenous photosensitizer in oral squamous cell carcinoma (OSCC), activating innate immunity and promoting antitumor responses.^101^ Conversely, Zhang et al. demonstrated enhanced therapeutic outcomes using HER-2-specific nanobody-photosensitizer conjugates with catalyst-binding complexes.^102^

### 5.2. Combination with photothermal therapy

Photothermal therapy (PTT) selectively ablates tumors via photothermal agents (PTAs) that convert near-infrared (NIR) light into localized hyperthermia. While valued for noninvasiveness and precision, PTT efficacy is hampered by inadequate PTA accumulation, tissue attenuation, and oxygen dependency. Bacterially-derived nanoplatforms offer innovative solutions.

For instance, Shuang et al. engineered calcium phosphate-coated *E. coli* outer membrane vesicles (OMVs) loaded with indocyanine green (ICG). Under NIR irradiation, these constructs elevated intratumoral temperatures and activated immune cells, significantly amplifying PTT effects.^103^ Similarly, Chen et al. developed *Shewanella* MR-1-based phototropic bacteria (*PTBs*) encapsulating methylene blue (MB), which inhibited mitochondrial function and suppressed heat shock protein (HSP) expression to potentiate PTT.^104^ To address thermal resistance, Wang et al. designed thermally activated biohybrids (TAB@Au) that accumulate at tumor sites, producing localized heat to induce cytolysin A (ClyA)-mediated lysis.^105^ Notably, VNP20009—a hypoxia-tropic *Salmonella* strain—was combined with polydopamine-mediated PTT, enhancing malignant melanoma eradication.^106^

Collectively, bacterially-enhanced phototherapy demonstrates potential to significantly enhance therapeutic indices through tumor-specific targeting and spatiotemporal control.

### 5.3. Combination with radiation therapy

Radiotherapy (RT) eradicates tumors by inducing DNA damage via ionizing radiation. However, off-target exposure risks damaging adjacent healthy tissues, causing acute/chronic toxicities. Moreover, radiotherapy-induced TME alterations—including vascular rarefaction and hypoxia—exacerbate radioresistance, thereby diminishing therapeutic efficacy.

Consequently, bacterially-enhanced radiotherapy represents a promising strategy to circumvent these limitations. Engineered bacteria can deliver radiosensitizing payloads or potentiate immune-mediated tumor clearance. Notably, *Listeria monocytogenes* has been repurposed as a vaccine vector for antigen delivery and immune stimulation. In seminal work, Hannan et al. demonstrated complete tumor regression in prostate cancer models using ADXS-31-142 (a *Listeria*-based vaccine) combined with RT.^107^ Subsequently, Qi et al. pioneered radioimmunotherapy by immobilizing ¹³¹I-labeled anti-PD-L1 antibodies on bacterial cellulose, which amplified immunogenic cell death (ICD) and activated diverse immune populations.^97^ Parallel innovations include radiolabeled inactivated vectors (*¹²⁵I-VNP/¹³¹I-VNP*) that achieve sustained intratumoral retention and target tumor-associated macrophages (TAMs).^108^ Furthermore, gut microbiota modulation may enhance colorectal cancer immunogenicity, radiosensitivity, and mitigate radiotoxicity—a frontier demanding exploration.^109^

### 5.4. Combination with chemotherapy drugs

Despite therapeutic advances, chemotherapy remains an oncological cornerstone, yet confronts dose-limiting toxicity, tumor heterogeneity, and multidrug resistance. Bacterially-mediated drug delivery leverages tumor tropism and hypoxic penetration to enhance precision and safety.^110^

Specifically, *Salmonella typhimurium* colonizes necrotic/hypoxic tumoral niches inaccessible to conventional therapies.^111^ Against multidrug-resistant tumors, engineered *Salmonella* delivered siRNAs targeting MDR1, effectively suppressing tumor growth.^112^ Similarly, *Staphylococcus aureus* cell wall-derived liposomal biomimetics (Bio-Bac) encapsulating doxorubicin (DOX@Bio-Bac) demonstrated synergistic antitumor activity.^113^ These platforms not only improve drug targeting but also remodel the immunologically suppressive TME.

Bacterially-derived minicells constitute ideal vehicles for chemotherapeutic encapsulation; their physicochemical properties enable versatile payload delivery.^114^ siRNA-loaded minicells enhance chemosensitivity, permitting dose reduction and treatment abbreviation.^115^ To ensure payload stability, covalent conjugation (e.g., amide bonding) facilitates controlled drug release at tumor sites.^116^Future designs should incorporate TME-responsive linkers to optimize spatiotemporal drug release, thereby maximizing therapeutic indices.

### 5.5. Combination with immune checkpoint inhibitors

The human gut microbiota, a complex ecosystem residing in the gastrointestinal tract, is increasingly recognized as a pivotal modulator of host immunity and a determinant of therapeutic responses in cancer.^117^ Beyond its local effects, the gut microbiome exerts systemic influence, shaping the tumor immune microenvironment and thereby affecting the efficacy of anticancer therapies, particularly immune checkpoint blockade (ICB).^118^ Specific commensal bacteria and their metabolites (e.g., short-chain fatty acids) have been mechanistically linked to enhanced antitumor T cell responses and improved outcomes with ICB.^119^ This foundational understanding positions the gut microbiota not merely as a bystander but as a viable therapeutic target. Strategies to modulate it, ranging from dietary interventions and probiotics to more precise engineered microbial therapeutics, represent a highly studied frontier for overcoming limitations of current immunotherapies.^120^

Building on this foundation, the gut microbiota has emerged as a critical determinant of response to ICB. ICB targeting CTLA-4 and PD-1/PD-L1 restores antitumor T cell function. Despite improved tolerability, its clinical utility is limited by response heterogeneity, frequent dosing requirements, and immune-related adverse events (irAEs). Compelling evidence implicates the gut microbiota in modulating ICB efficacy.^121^ For example, Park et al. identified commensal suppression of PD-L2/RGMb signaling as a mechanism for enhanced antitumor immunity.^122^ Furthermore, *Enterococcus spp.* potentiated ICB responses; Joachim et al. demonstrated that deaminotyrosine (DAT) amplifies type I interferon signaling and remodels the microbiota to overcome resistance.^123^ Synthetic biology enables engineered probiotics to locally deliver checkpoint inhibitors within the TME, minimizing systemic exposure while maximizing intratumoral concentrations. Danino's group pioneered probiotic-guided CAR-T cell therapy: *E. coli Nissle 1917* secreting synthetic antigens and CXCL11 recruits and activates systemically administered CAR-T cells within tumors, overcoming poor infiltration and immunosuppression characteristic of solid tumors.^124^

Moreover, photo-activatable bacterial vectors can achieve spatiotemporally controlled release of checkpoint inhibitors. Bacteria modified by internal and external modifications can produce a localized thermal effect under light irradiation and simultaneously release immune checkpoint inhibitors to trigger immune activation.^125^ This precision targeting elevates the therapeutic index of ICB and provides a rational platform for combination regimens.

### 5.6. Combination with oncolytic virotherapy

As described in Section 4.4, oncolytic viruses selectively infect and lyse tumor cells while eliciting systemic antitumor immunity. This section focuses on the synergistic co-administration of oncolytic viruses with tumor-targeting bacteria as a distinct combination paradigm. Herpesviridae have become a predominant focus in OVT development due to their inherent oncotropism. Specific strains exhibit intrinsic tumor selectivity, while others have been genetically engineered to enhance tumor-specific targeting.^126^ In this combinatorial approach, bacteria and viruses function as co-therapeutics. For instance, the immunomodulatory properties of bacteria can be harnessed to reshape the tumor microenvironment, thereby potentially overcoming residual antiviral defenses and creating a more permissive niche for viral replication. For example, Xie et al. demonstrated that herpesvirus-mediated delivery of the E3 ubiquitin ligase IpaH9.8 effectively degraded GBP1, resulting in significant antitumor efficacy in vivo.^127^ When such viral strategies are combined with tumor-colonizing bacteria, the local immune activation and enhanced intratumoral distribution mediated by bacteria can synergistically amplify the overall therapeutic outcome.

Nevertheless, bacterial–OV synergistic therapy faces translational challenges. Precise spatial–temporal control of both bacterial and viral dissemination is essential to prevent collateral damage to healthy tissues. Modulation of the compounded immune responses is also required to mitigate adverse events, and maintaining the stability and predictable interaction of both vectors in vivo remains a critical technical barrier to therapeutic efficacy.

### 5.7. Combination with other cancer therapies

The convergence of bacterial immunotherapy and ferroptosis induction represents an emerging anticancer paradigm. Ferroptosis—characterized by iron-dependent phospholipid peroxidation—potentiates antitumor immunity through dendritic cell activation by damage-associated molecular patterns (DAMPs) released during ferroptotic cell death. This cascade promotes CD8⁺ T cell cross-priming and IFN-*γ* production, establishing a proimmunogenic milieu. Cai et al. elucidated that ferroptosis induction overcomes resistance to immunotherapeutics.^128^ Furthermore, photodynamic therapy synergizes with ferroptosis inducers by exploiting Fenton reaction-derived reactive oxygen species to amplify cytotoxic effects.^129^ Bacterial vectors can be engineered to deliver ferroptosis triggers with tumor specificity, enhancing tumoricidal activity while stimulating robust systemic immunity.

Notably, engineered *Salmonella SL7207* displaying the HPV E7 antigen (9RE7) on its outer membrane—designated *Sal-9RE7*—significantly enhanced immunogenicity and antitumor efficacy when co-administered with the albumin-IFNβ (Alb-IFNβ) adjuvant.^130^ Therefore, adjuvants are posited to critically augment anticancer immunotherapy, particularly in in situ tumor vaccine formulations designed to optimize immune activation efficiency.^131^ These findings collectively demonstrate that bacterial immunotherapy combined with adjuvant approaches may enhance therapeutic indices, mitigate adverse effects, and potentiate adaptive immune responses, offering novel translational avenues for oncology.

## 6. The clinical trials of bacteria-based cancer immunotherapy

Bacteria-based cancer immunotherapies are undergoing extensive clinical evaluation, primarily leveraging four microbial vectors (Table 2). Notably, intravesical *Mycobacterium bovis* BCG remains the first-line therapy for non-muscle-invasive bladder cancer (NMIBC), supported by its established tolerability and safety profile. Its genetically attenuated derivative, VPM1002BC, has shown improved therapeutic responses in clinical trials. Combination regimens pairing BCG with agents such as the IL-15 superagonist *N*-803, as well as novel approaches like nadofaragene firadenovec and the nano-immunotherapy OncoTherad (MRB-CFI-1), have demonstrated encouraging efficacy in BCG-unresponsive NMIBC cohorts.^132^

**Table 2.** Clinical Trials of Bacteria-Based Therapies for Various Cancer Types.

Bacteria type	Cancer type	Test Methods	Test results	Clinical phase	Clinical Number	Ref
BCG Natural BCG strain (without genetic modification)	Non-muscle invasive bladder cancer	Durvalumab group (intravenous injection); D + BCG group (intravesical injection); D + EBRT group (radiotherapy)	Durvalumab + EBRT Complete Response (CR): 33% (at 12 months) Durvalumab + BCG CR: 73% (at 12 months)	Phase 1	NCT03317158	[133]
BCG VPM1002BC [Recombinant strain expressing *Listeria* hemolysin O (hly)]	Non-muscle invasive bladder cancer	Multiple intraperitoneal injections	VPM1002BC:After 1 year, 49.3% were recurrence-free	Phase 1/Phase 2	NCT02371447	[134]
BCG Natural BCG strain (without genetic modification)	Non-muscle invasive bladder cancer	Intravenous multiple injections	Atezolizumab CR: 33% Atezolizumab + BCG CR: 42%	Phase 1/Phase 2	NCT02792192	[135]
*Escherichia coli* Engineering strains expressing HPV16/18 L1 protein	cervical cancer	Multiple intramuscular injections	per-protocol susceptible population (PPS-E) preventive efficacy: 100.0% per-protocol susceptible population (PPS-PI) preventive efficacy: 97.8%	Phase 3	NCT01735006	[136]
*Escherichia coli* Engineering strains expressing HPV16/18 L1 protein	cervical cancer	Multiple intramuscular injections	Different vaccination intervals had little effect on the immunogenicity of the HPV vaccine produced by ***E. coli***, which was stable.	Phase 3	/	[137]
*Listeria monocytogenes* ADXS31-142 [Prostate-specific antigen (PSA) expression]	PCa(prostatic cancer)	Multiple injections intravenously	ADXS31-142 Median Overall Survival (OS): 7.8 months ADXS31-142 + Pembrolizumab OS: 33.7 months	Phase 1/Phase 2	NCT02325557	[138]
*Listeria monocytogenes* CRS-207 [Expression of mesothelin]	pancreatic cancer	Multiple injections intravenously	Arm A (with Nivolumab) OS: 5.9 months Arm B (without Nivolumab) OS: 6.1 months	Phase 2	NCT02243371	[139]
*Listeria monocytogenes* ADXS-HPV [Expression of HPV-16 E7 antigen]	cervical cancer	Multiple injections intravenously	The 12-month OS rate for ADXS-HPV was 38%, which is efficacious and tolerable.	Phase 2	NCT01266460	[140]
*Listeria monocytogenes* CRS-207 [Expression of mesothelin]	pancreatic cancer	Multiple injections intravenously	Patients with higher abundance of CD8 + CD45RO- CCR7- CD57 + cells and lower abundance of CD14 + CD33 + CD85j + cells had improved overall survival.	Phase 2	NCT02004262	[141]
*Salmonella* typhimurium Saltikva [Expression of human leukocyte IL-2]	gastric carcinoma	Single oral dose	NK cells increased from an average of 12.1% to 14.6% NK-T cells increased from an average of 3.4% to 5.6%	Phase 1	/	[142]
*Clostridium novyi* NT strain [Naturally attenuated strain (*α*-toxin gene removed)]	solid tumor	Single injection within the tumor	Stable Disease (SD) : 86% Progressive Disease (PD) : 13%	Phase 1	NCT01924689	[143]
Bifidobacterium CBM588(without genetic modification)	renal carcinoma	Navulizumab and ibritumomab: Multiple injections intravenously CBM588: Daily oral administration	CBM588 prolongs progression-free survival (PFS) and increases Bifidobacterium abundance in mRCC patients.	Phase 1	NCT03829111	[144]
Lactobacillus BLS-M07 [Expression of HPV-16 E7 antigen]	High-grade cervical intraepithelial neoplasia 3 (CIN 3)	Multiple oral doses	BLS-M07 was effective in treating CIN 3, with 75% of patients improving.	Phase 1/Phase 2	NCT02195089.	[145]
Lactobacillus IGMKK16E7 [Expression of HPV-16 E7 antigen]	HPV-16 positive high-grade cervical intraepithelial neoplasia 2 and 3 (CIN 2 and CIN 3)	Multiple oral doses	High dose group CR: 31.7% Placebo group CR: 12.5% High-dose group CR: 40.0% in HPV-16 single-positive patients Placebo group CR: 12.5%	Phase 1/Phase 2	jRCT2031190034	[146]
Probiotics Combined probiotics(without genetic modification)	Relapsed/refractory multiple myeloma (MM); Acute lymphoblastic leukemia (ALL); ​​​​​​​Non-hodgkin Lymphoma (NHL)	Multiple administration	Composition and Function of the Gut Microbiome Correlate with Efficacy of CAR-T Therapy and Severity of CRS(Bifidobacterium, Faecalibacterium, etc)	/	ChiCTR1800017404	[147]

Significant progress has also been achieved with engineered *Escherichia coli* vectors, particularly in HPV vaccinology. An *E. coli*–produced bivalent HPV-16/18 vaccine achieved 100% efficacy against HPV-16/18-associated high-grade genital lesions in Phase III trials, demonstrating robust immunogenicity, favorable safety, and sustained clinical protection despite minor schedule deviations.^136^ This platform offers substantial potential to enhance global vaccine accessibility.

Attenuated *Listeria monocytogenes* strains have emerged as promising candidates for treating pancreatic adenocarcinoma and metastatic castration-resistant prostate cancer (mCRPC).^138^ ^,^ ^139^ In parallel, obligate anaerobes such as the non-toxic *Clostridium novyi*-NT spores have shown promise in trials by selectively germinating in hypoxic tumor cores, inducing oncolysis and local immune responses in advanced solid tumors.^78^ ^,^ ^143^ Finally, probiotic organisms exert antitumor effects through gut microbiome modulation and function as vaccine delivery vehicles. Clinical evidence suggests that strains such as *Clostridium butyricum CBM588* and *Bifidobacterium lactis BLS-M07* can potentiate responses to immune checkpoint inhibitors and CAR T-cell therapies, underscoring their value as adjuvants in multimodal oncology regimens.^144^

Collectively, these clinical advances highlight the transformative potential of bacterial immunotherapies, though broader clinical implementation faces challenges including precise control of bacterial viability in vivo, managing potential systemic toxicity, heterogeneous patient responses, and complex manufacturing and regulatory pathways for live biotherapeutic products. Ongoing trials continue to refine safety, dosing, and efficacy across diverse malignancies, positioning these strategies as an emerging pillar in the oncologic treatment paradigm.

## 7. The translational challenges and opportunities of bacteria-based cancer immunotherapy

Synthetic biology breakthroughs over the past decade have transformed the landscape of bacterial cancer immunotherapy, enabling engineered vectors to selectively proliferate within the TME and suppress neoplastic growth.^148^ However, this rapid progress is tempered by translational hurdles, including innate bacterial cytotoxicity, manufacturing complexities, and unresolved clinical safety-efficacy paradigms.

The primary challenge lies in mitigating inherent pathogenicity during bacterial vector engineering. Attenuation strategies often employ CRISPR-Cas9–mediated inactivation of virulence determinants, as exemplified in the development of *Listeria monocytogenes* and *Salmonella enterica* serovar Typhimurium vaccines. These approaches reduce pathogenic potential while preserving or enhancing antitumor efficacy.^149^ Furthermore, synthetic biology enables the engineering of bacteria resilient to physiological stressors, thereby improving therapeutic durability. Raman et al. exemplified this strategy by systematically integrating bacterial chassis, therapeutic payloads, delivery mechanisms, immunomodulatory elements, and genetic circuitry, establishing novel anticancer platforms.^150^

Concurrently, manufacturing scalability and quality assurance remain significant translational challenges. To enhance production efficiency and product quality, researchers have implemented advanced bioreactor technologies, multi-step purification processes, and stringent quality control measures, including comprehensive safety assessments and molecular dynamics simulations. These strategies aim to ensure both safety and efficacy throughout the manufacturing pipeline. Developing an efficient production system requires the careful selection of bacterial strains, balancing attenuation with therapeutic potency, and leveraging bioreactor platforms to achieve high-density cultures. Recent advances, such as microfluidic chemotaxis-based devices, have further improved the screening of bacterial candidates with strong tumor-targeting capabilities.^151^ Quality control is equally critical. Robust safety evaluations—including toxicology, immunogenicity, and sensitization assays—are essential to confirm compliance with established criteria for bacterial enumeration, purity, and activity. In addition, molecular dynamics simulations and 3D structural prediction tools facilitate the assessment of vaccine constructs for structural stability and immunogenicity.^152^ From a cost perspective, process optimization and technological innovation remain key to reducing manufacturing expenses without compromising quality. A notable example is the STING-targeting bacterial immunotherapeutic developed by Daniel S. Leventhal et al., which was designed to meet regulatory standards while minimizing synthesis costs.^153^

Notably, clinical implementation faces multifaceted challenges. Preclinical safety evaluation must address both immunogenicity profiling and ecotoxicological risk assessment.^154^ Although strategies such as type III secretion system–mediated effector delivery can enhance antitumor potency,^155^ clinical outcomes often fail to replicate preclinical efficacy. A well-documented example is *Salmonella VNP20009*, which exhibited an acceptable safety profile but demonstrated limited therapeutic benefit due to suboptimal tumor colonization and rapid immune clearance.^156^ Administration route optimization adds another layer of complexity. Intravenous injection carries the risk of systemic dissemination, intratumoral delivery is constrained by anatomical accessibility, and oral administration must overcome gastrointestinal degradation. Nanotechnology-based encapsulation offers a promising solution by improving bacterial stability and tumor-targeting specificity, particularly in pulmonary malignancies.^157^ Similarly, dose-limiting toxicities—such as systemic inflammatory responses observed in BCG-treated bladder cancer—may be mitigated by incorporating bioactive adjuncts,^158^ underscoring the need for precise dose and frequency optimization to maximize therapeutic indices.

Despite these hurdles, interdisciplinary collaboration continues to drive innovation. Advances in synthetic biology, biomaterials, and immunoengineering, combined with rigorous clinical evaluation, position bacterial immunotherapy as a potential cornerstone of future cancer treatment, offering the prospect of highly specific and durable tumor eradication.

## 8. Outlooks

The convergence of synthetic biology and bacterial immunotherapy represents a paradigm shift in oncology, offering substantial therapeutic promise. Advances in bioengineering and nanotechnology have driven the development of tumor-targeted engineered bacteria capable of disrupting metabolic pathways and eliciting robust antitumor immune responses. This emerging field introduces innovative translational strategies, including microbiota-based interventions, multimodal combination therapies, and utilization of bacterial components as therapeutic agents.Microbiota engineering—empowered by synthetic biology—enables precise genetic modification of microbial vectors, transforming them into localized biofactories for in situ delivery of immunotherapeutic payloads such as nanobodies, PAMPs, STING agonizts, and cytokines. However, clinical translation of STING agonizts remains limited by metabolic instability and off-target immune activation. To address these challenges, nano-agonist formulations and engineered probiotics have been developed to enhance targeting specificity and improve safety, thereby facilitating clinical application. Despite encouraging preclinical data, successful clinical implementation requires rigorous safety validation, efficacy confirmation within the TME, and establishment of GMP-compliant manufacturing pipelines for large-scale production.^42^ Importantly, environmentally responsive bacterial vectors enable tumor-selective drug delivery through sensing of TME cues. Combination strategies integrating bacterial immunotherapy with photothermal, photodynamic, radiotherapeutic, or chemotherapeutic adjuvants can overcome resistance mechanisms and enhance overall efficacy. Collectively, these integrated approaches offer solutions to the challenges of tumor heterogeneity and acquired resistance, advancing next-generation multimodal cancer treatment paradigms.

Ultimately, synthetic biology-driven innovations pave the way for highly targeted, efficient, and personalized cancer therapeutics. Continued interdisciplinary collaboration will be essential to overcome translational hurdles. With sustained innovation and clinical validation, bacterial immunotherapy is poised to become a cornerstone in the oncologic armamentarium, delivering transformative therapeutic options for cancer patients.

## Data Availability

Data sharing is not applicable to this article as no data were created or analyzed in this research.
